# Therapeutic Potential of Targeting the PERK Signaling Pathway in Ischemic Stroke

**DOI:** 10.3390/ph17030353

**Published:** 2024-03-08

**Authors:** Xinyuan Yu, Lihong Dang, Ran Zhang, Wei Yang

**Affiliations:** Multidisciplinary Brain Protection Program, Department of Anesthesiology, Duke University Medical Center, Box 3094, 303 Research Drive, Durham, NC 27710, USA

**Keywords:** ER stress, UPR, ischemia, autophagy, oxidative stress, mitochondria

## Abstract

Many pathologic states can lead to the accumulation of unfolded/misfolded proteins in cells. This causes endoplasmic reticulum (ER) stress and triggers the unfolded protein response (UPR), which encompasses three main adaptive branches. One of these UPR branches is mediated by protein kinase RNA-like ER kinase (PERK), an ER stress sensor. The primary consequence of PERK activation is the suppression of global protein synthesis, which reduces ER workload and facilitates the recovery of ER function. Ischemic stroke induces ER stress and activates the UPR. Studies have demonstrated the involvement of the PERK pathway in stroke pathophysiology; however, its role in stroke outcomes requires further clarification. Importantly, considering mounting evidence that supports the therapeutic potential of the PERK pathway in aging-related cognitive decline and neurodegenerative diseases, this pathway may represent a promising therapeutic target in stroke. Therefore, in this review, our aim is to discuss the current understanding of PERK in ischemic stroke, and to summarize pharmacologic tools for translational stroke research that targets PERK and its associated pathways.

## 1. Introduction

Ischemic stroke, the most prevalent stroke type, is associated with a high risk of mortality and long-term disabilities [1]. Apart from acute reperfusion therapy, treatment options for stroke patients are limited. This underscores the urgency of developing new and effective stroke therapeutics that can mitigate brain damage and improve functional recovery. To this end, a thorough understanding of stroke pathophysiology is key.

After ischemic stroke, massive cell death and blood–brain barrier (BBB) damage lead to a profound change in the extracellular environment of the affected brain regions. In response to this change, many pathways are activated in brain cells, including both pro-survival and pro-death pathways, which represent potential therapeutic targets for stroke treatment. One such pathway is the unfolded protein response (UPR), which is initiated in the endoplasmic reticulum (ER). The ER is the primary site in cells for protein translation, modification, folding, and maturation, as well as calcium buffering [2]. Thus, ER function is critical for cellular protein homeostasis (proteostasis) [2,3]. After stroke, the adverse environment disrupts calcium homeostasis and exposes cells to toxic reactive oxygen species (ROS). Coupled with reduced energy availability, ER function related to proteostasis is impaired after stroke, leading to the accumulation of misfolded or unfolded proteins in the ER—a state known as ER stress [4,5,6]. To restore ER function and reestablish cellular proteostasis, mammalian cells have three major adaptive response pathways mediated by ER stress sensors: ATF6 (activating transcription factor 6), IRE1 (inositol-requiring enzyme 1), and PERK (protein kinase RNA-like ER kinase) [2]. These pathways are branches of the UPR. 

A wealth of evidence indicates that stroke causes ER stress and activates the UPR in the brain [4,5,6]. However, the effect of UPR activation in stroke has not been fully established, as data supporting both its detrimental and beneficial roles have been reported. This discrepancy is likely attributed to the interpretation of stroke data related to the PERK pathway. Unlike the ATF6 and IRE1 UPR branches, which are predominantly considered protective in cells under ER stress, the PERK UPR branch can either restore cellular homeostasis or activate cell death processes [7,8,9,10]. In the early phase of ER stress, activation of all three UPR branches leads to the suppression of general protein synthesis, the improvement of ER capacity for protein folding, and enhanced clearance of unfolded proteins, collectively mitigating ER stress and promoting cell survival. However, if ER stress persists and the activated UPR fails to restore ER function, cell death processes ensue, and PERK signaling plays a major role in these processes [7]. Notably, the PERK pathway has been increasingly studied as a therapeutic target for aging-related cognitive decline and neurodegenerative diseases [11,12]. Thus, a better understanding of the role of PERK in stroke outcomes is critically important to the stroke field. In this review, we first discuss the primary cellular pathways/processes that are regulated by PERK signaling, and then summarize key experimental findings about PERK in ischemic stroke. 

## 2. General Overview of the UPR in Ischemic Stroke

UPR activation exerts its effects by modulating both transcriptional and translational programs in cells [2]. Upon sensing ER stress, ATF6 translocates from the ER to the Golgi where it is cleaved to generate the short-form ATF6 (sATF6), while IRE1 becomes an endoribonuclease that mediates the non-conventional splicing of X-box binding protein 1 (XBP1) mRNA, subsequently generating the active XBP1s protein. Both sATF6 and XBP1s are potent transcriptional factors and, once in the nucleus, upregulate expression of many genes related to protein folding, maturation, and degradation, such as ER chaperones GRP78 (or BiP), GRP94, and ERAD-related proteins [2]. Notably, XBP1s can also upregulate expression of the enzymes involved in the hexosamine biosynthetic pathway (HBP) and thus increase global O-GlcNAcylation [13,14,15]. This post-translational modification has been demonstrated to be a pro-survival response under various stress conditions [16].

Using mice with global deletion of *Atf6 (Atf6^−/−^*), a previous study showed that these mice exhibited worse brain damage after stroke [17]. Our group has developed a conditional and tamoxifen-inducible sATF6 knock-in mouse line, sATF6-MER [18]. After generating sATF6-MER;Emx1-Cre (sATF6-KI^Neuron^) mice with neuron-specific expression of sATF6 in the brain, we subjected these mice to transient filament middle cerebral artery occlusion (tMCAO), a stroke model, and found that forced activation of the ATF6 branch in neurons significantly reduced infarct volumes and improved neurologic function [18]. Recently, we examined sATF6-KI^Neuron^ mice in permanent stroke using photothrombotic (PT) and transcranial MCAO models and observed improved short- and long-term stroke outcomes in these mice [19]. Together, the current data support the notion that activation of the ATF6 branch is protective in ischemic stroke. 

Leveraging a gain-of-function mouse model (TRE-XBP1s;Camk2a-tTA [XBP1s-TG^Neuron^]) and a loss-of-function mouse model (*Xbp1^f^*^l/fl^;Emx1-Cre [Xbp1-cKO^Neuron^]), our group has provided the first evidence that activation of XBP1 signaling in neurons is neuroprotective in both transient and permanent ischemic stroke models, and that activation of the XBP1/HBP/O-GlcNAc axis is a critical mechanism that underpins XBP1-mediated neuroprotection [14,15]. In support of this mechanism, we also found that pharmacologically boosting O-GlcNAcylation with thiamet-G partially reversed worse stroke outcomes observed in Xbp1-cKO^Neuron^ mice [15]. Consistently, it has been shown that *Xbp1s* overexpression in cardiomyocytes protects the heart from ischemia/reperfusion injury, reducing the infarct area by nearly 50% [13]. Of note, however, a recent study reported that sustained overactivation of XBP1s signaling in neurons can cause seizure and animal death [20]. Thus, careful consideration is needed for long-term treatments that target IRE1/XBP1 signaling. 

Under ER stress, activated PERK phosphorylates serine 51 of the α subunit of eukaryotic translation initiation factor 2 (eIF2) which leads to attenuation of global protein synthesis, as detailed below. The consequence is a reduction in ER workload, which facilitates the restoration of ER homeostasis. An increase in phosphorylated eIF2α (p-eIF2α) and protein synthesis inhibition (PSI) has been found in the ischemic brain [21,22,23]. However, this observation alone does not necessarily indicate that the PERK branch is the cause of stroke-induced PSI, because four kinases—GCN2, PKR, HRI, and PERK—have been identified as eIF2α kinases [8]. These kinases are differentially activated according to stress types: GCN2 responds to nutrient deprivation, PKR to double-stranded RNA, HRI to low heme concentration, and PERK to ER stress. Using global *Perk* knockout mice, an early study suggested that PERK is responsible for post-ischemic eIF2α phosphorylation [24]. Further supportive data regarding the involvement of the PERK pathway in stroke-induced PSI come from experiments on *Perk^f/f^*;Camk2a-Cre (PERK-cKO^Neuron^) mice in which *Perk* is specifically deleted in neurons [21]. In these conditional knockout mice, ischemia-induced p-eIF2α is markedly suppressed, and levels of protein synthesis in the brain are higher in PERK-cKO^Neuron^ vs. control mice. Moreover, salubrinal, an inhibitor of p-eIF2α de-phosphorylation, can largely reverse the elevated protein synthesis observed in PERK-cKO^Neuron^ mice [21]. All these data together support a mechanistic link between PERK, p-eIF2α activation, and PSI in the ischemic brain. Moreover, PERK-cKO^Neuron^ mice have worse acute stroke outcomes [21]. However, it must be noted that although the current data indicate that a protein synthesis-related mechanism drives the effects of PERK signaling on stroke outcomes, other PERK-controlled downstream pathways/processes may also be involved, as discussed below. 

## 3. PERK Signaling

**Figure 1:**
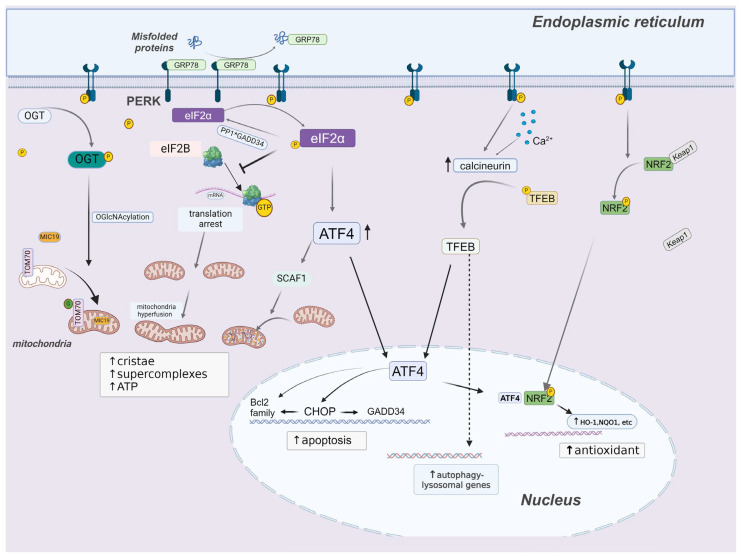
PERK-related pathways and processes. Upon ER stress, GRP78 dissociates from PERK, leading to PERK activation via autophosphorylation. Phosphorylated PERK acts as a kinase, primarily targeting eIF2α. Phosphorylation of eIF2α has several consequences, including the suppression of global protein synthesis, facilitation of mRNA translation for selective genes (e.g., ATF4), and mitochondria remodeling. Activated PERK can also phosphorylate OGT and NRF2, thereby regulating mitochondrial and redox homeostasis, respectively. Moreover, PERK may interact with calcineurin to modulate the autophagy–lysosomal pathway.

PERK is a transmembrane protein that features a luminal ER stress-sensing domain and a cytosolic kinase domain. According to the current activation model, PERK remains an inactive state when chaperon GRP78 is bound to its luminal domain. Upon ER stress, GRP78 dissociates from PERK to engage with unfolded proteins. This activates PERK by promoting its dimerization and autophosphorylation [2]. While its primary substrate is eIF2α, PERK can phosphorylate other signaling factors including nuclear factor erythroid 2-related factor 2 (NRF2). Importantly, PERK also exerts functions that are independent of its kinase activity [11,12]. Major PERK-related signaling pathways/processes are illustrated in Figure 1.

### 3.1. PERK and p-eIF2α

PERK primarily phosphorylates eIF2α, a subunit of eIF2, which consists of α-, β-, and γ-subunits. eIF2 is a GTP binding protein that is central to the mRNA translation process. In the translation initiation process, Met-tRNA, eIF2γ, and GTP combine to form the functional ternary complex (TC). The TC scans the mRNA, along with other initiation factors. When the initiator Met-tRNA successfully pairs with the AUG start codon, GTP undergoes hydrolysis, resulting in the release of eIF2•GDP from the TC. To reassemble the TC, GDP is displaced by GTP with the assistance of the guanine nucleotide exchange factor, eIF2B [25]. However, phosphorylated eIF2α can strongly bind to eIF2B, prevent GTP from reloading into the TC, and thus arrest global protein synthesis [2,26]. Interestingly, the presence of p-eIF2α favors selective translation of mRNAs containing short upstream open reading frames (uORFs) in the 5′-UTR of genes among which transcription factor 4 (ATF4) has been most studied. ATF4 regulates expression of a range of genes involved in redox control, protein folding, and amino acid metabolism to facilitate restoration of cell homeostasis [2,26]. Critically, PERK-induced p-eIF2α is essential to the survival of cells under ER stress as it reduces ER workload [27]. For example, salubrinal increases levels of p-eIF2α, reduces translation rates, and protects cells from ER stress [28].

### 3.2. PERK and Autophagy

TEFB and TFE3 are transcription factors that play a critical role in the autophagy pathway [29]. Under ER stress, both TFEB and TFE3 are activated in a PERK-dependent manner, and their activation promotes expression of autophagic and lysosomal genes, thereby linking ER stress and autophagy [30]. Mechanistically, PERK may interact with calcineurin (a Ca^2+^-dependent phosphatase). This interaction results in the release of Ca^2+^ from the ER to the cytosol, which enhances the activity of calcineurin. Calcineurin, in turn, dephosphorylates TFEB, promoting its translocation into the nucleus where TFEB initiates expression of autophagy-lysosomal components [31,32]. Importantly, a large body of evidence indicates that activation of autophagy protects the brain against ischemic stroke [33]. 

### 3.3. PERK and Redox Homeostasis

ROS are byproducts of various cellular processes including mitochondrial respiration and protein folding. Excessive ROS can damage macromolecules such as nucleic acids and proteins, posing a threat to cellular health. When ROS cannot be effectively cleared by the cellular antioxidant system, oxidative stress occurs. Mounting evidence indicates that PERK signaling regulates oxidative homeostasis, primarily by phosphorylating NRF2 [34]. NRF2, a master transcription factor, can mitigate the harmful effects of ROS by controlling expression of many antioxidant genes including heme oxygenase-1 (HO-1) [35]. Under physiologic conditions, NRF2 binds to the aptamer of Cullin 3-based E3 ubiquitin ligase, known as Kelch-like ECH-associated protein 1 (Keap1), which inhibits NRF2 nuclear import and promotes its ubiquitination and subsequent degradation [36]. Once phosphorylated, NRF2 dissociates from Keap1 and translocates to the nucleus where it upregulates expression of antioxidant genes by binding to the antioxidant response element (ARE) in the promoter region. There is also evidence that ATF4 interacts with NRF2 to regulate HO-1 gene expression [37]. Notably, oxidative stress is a main component of stroke pathophysiology, and NRF2 signaling is critical to restoring redox homeostasis and protecting the brain from stroke injury [35]. It has been shown that *Nrf2* knockout mice had larger infarct sizes and worse functional outcome after 90 min MCAO [38]. Consistently, transgenic mice overexpressing HO-1 in the brain exhibited significantly smaller infarct sizes [39]. Thus, the worse stroke outcome observed in *Perk* knockout mice may also be partially due to increased oxidative damage [21].

### 3.4. PERK and Mitochondrial Homeostasis

Often, stressors that cause ER stress also lead to mitochondrial dysfunction, characterized by increased ROS, decreased ATP production, and impaired mitochondrial protein folding [11]. Indeed, stroke induces severe metabolic stress on brain cells, resulting in both ER stress and mitochondrial dysfunction [40]. Importantly, the intricate crosstalk between the ER and mitochondria is well documented, with physical contacts known as mitochondria–ER-associated membranes (MAMs) that facilitate signal exchange via Ca^2+^ flux and signaling proteins residing at the contact sites [41]. PERK has been found in ER MAMs, and PERK-deficient cells exhibit decreased ER–mitochondrial contact [42]. Notably, a substantial body of evidence underscores the crucial role of PERK in preserving and restoring mitochondrial homeostasis, which involves multiple mechanisms, including modulation of translational and transcriptional programs and mitochondrial remodeling [41]. This role is critically important, as mitochondria are the powerhouse for cellular functions as well as the main source of ROS in cells. Failure to restore mitochondrial homeostasis may lead to energy deficiency and increased ROS production, further aggravating ER stress.

In response to mitochondrial dysfunction, a set of mitochondrial protective genes, including chaperone and protease genes, is upregulated. This is largely mediated by the transcription factor ATF5 [43]. Interestingly, ATF5 mRNA transcripts contain uORFs and, like the ATF4 gene, its translation is increased when eIF2α is phosphorylated under ER stress [44]. Thus, the PERK/eIF2α pathway contributes to restoring mitochondrial homeostasis by enhancing the ATF5-reguated mitochondrial protective program. 

In response to energic and metabolic demands under stress conditions, adaptive remodeling of mitochondrial morphology and function occurs. One adaptive response to nutrient deprivation is the elongation and hyperfusion of mitochondria, which aims to maintain ATP production and sustain cell viability [45]. This response involves the PERK pathway. In a cell culture study, the investigators used CRISPR-Cas9 to delete XBP1, PERK, and ATF6 individually, and found that only PERK deletion ablated the increase in supercomplex levels and compromised oxidative phosphorylation-dependent ATP production after glucose deprivation [46]. Mechanistically, it has been reported that the PERK/eIF2α/ATF4 axis can mediate upregulation of supercomplex assembly factor 1 (SCAF1) [46]. However, ER stress-induced mitochondrial hyperfusion is related to downstream PERK-induced translation attenuation, but not ATF4 [47]. Of note, hyperfusion is a protective mechanism to increase cellular energetic capacity and promote cell recovery from stress. As expected, genetic disruption of PERK-regulated hyperfusion increases mitochondrial fragmentation and makes cells more sensitive to ER stress. Another adaptive effect mediated by PERK is to increase mitochondrial cristae densities, which boosts oxidative phosphorylation and may also have antiapoptotic effects by preventing cytochrome C release [48]. Mechanistically, PERK phosphorylates OGT, which then mediates O-GlcNAcylation of translocase of the outer membrane 70 (TOM70). This post-translational modification enhances TOM70-assisted MIC19 mitochondrial protein import and consequently, increases cristae biogenesis and cell respiration [49]. Moreover, PERK is implicated in mitochondrial lipid homeostasis. A recent study showed that E-Syt1 interacted with PERK to serve as a molecular component of the lipid trafficking machinery at MAMs, but this role seems independent of PERK function in the UPR [50].

### 3.5. PERK and Cell Death

In cases of prolonged ER stress, cells may undergo apoptosis-mediated cell death through two main pathways: the mitochondrial pathway and the death receptor pathway [7]. The mitochondrial pathway of apoptosis is largely mediated by Bcl2 family members, including pro-apoptotic (e.g., Bim and Bax) and anti-apoptotic (e.g., Bcl2) factors. Many of these genes can be regulated by ATF4 or transcription factor CCAAT/enhancer-binding protein homologous protein (CHOP/GADD153). Notably, CHOP expression can be upregulated by ATF4. Thus, the PERK/eIF2α/ATF4/CHOP axis is commonly mentioned in the literature.

Studies have shown that CHOP overexpression downregulates Bcl2 and upregulates Bim to promote mitochondria-mediated apoptosis under ER stress [51,52]. This process involves the formation of pores in mitochondria regulated by Bcl2 family proteins, which results in the release of cytochrome C and subsequent activation of the caspase cascade. Another proposed mechanism by which CHOP induces apoptosis is that CHOP promotes the reversal of translational repression, thus overloading the ER during ER stress [53]. This effect is attributed to CHOP-mediated upregulation of growth arrest and DNA damage-inducible 34 (GADD34). GADD34, as a regulatory subunit, together with protein phosphatase-1 (PP1), forms a phosphatase complex that specifically dephosphorylates p-eIF2α, thereby reversing PSI and increasing protein synthesis [54,55]. Further, CHOP can upregulate expression of death receptor 5 (DR5), which interacts with death ligands to trigger apoptosis. Indeed, CHOP has been widely considered a key pro-apoptotic mediator in ER stress-induced cell death [56,57]. Of note, however, overexpression of CHOP alone does not cause apoptosis [58], and CHOP also activates pro-survival autophagic genes [59]. Importantly, a recent study provided compelling evidence showing that CHOP promoted stress resolution and subsequent proliferation, which conferred a beneficial adaptive role under mild ER stress [60]. Therefore, an increase in CHOP expression should not always be interpreted as a detrimental response. 

Collectively, the current literature indicates that in ER-stressed cells, the PERK branch plays a dual role. In the early phase of ER stress, it acts in a pro-survival manner by reducing ER workload, upregulating ER stress-resolving factors, and restoring mitochondrial homeostasis. However, in the late phase, if ER homeostasis is not re-established, the PERK branch can switch to a pro-apoptotic role.

## 4. The PERK Pathway in Ischemic Stroke

The role of the PERK pathway in stroke outcomes appears controversial in the literature. As discussed above, this is likely due to the dual functions of this pathway, which complicates data interpretation. The detrimental role of PERK activation in stroke outcomes is often implicated by pharmacologic studies in which protective effects are associated with reduced levels of PERK branch components such as p-eIF2α, ATF4, and CHOP [61,62,63]. However, there is a clear caveat in interpreting cause and effect in such studies. The extent to which the UPR is activated is associated with the severity of the ER stress, which, in this case, is related to stroke damage. Thus, if the treatment under investigation protects the brain from stroke damage, it is expected that UPR markers are activated to a lesser extent, compared to the control group. Therefore, caution is warranted when concluding that the protective effect of the treatment is caused by suppression of the UPR or ER stress. Importantly, stroke studies using either UPR-specific mouse tools or compounds provide strong evidence that activation of the UPR provides beneficial effects during the acute stroke phase. However, it must be noted that the involvement of PERK in the stroke recovery phase remains largely unexplored. 

### 4.1. Experimental Evidence from PERK-Specific Mouse Models

Two studies have used conditional PERK knockout mouse models in stroke research, and both support the notion that activation of the PERK branch during the acute phase is brain protective in stroke [21,64]. 

The study from our group was focused on the PERK pathway in neurons, using neuron-specific *Perk* knockout (PERK-cKO^Neuron^) mice and a transient MCAO stroke model. Our data indicated that deletion of *Perk* in neurons reduced p-eIF2α levels in the brain, and significantly worsened the neurologic outcomes after stroke [21]. In a more recent study, the authors crossbred *Perk*^f/f^ mice with GFAP-Cre mice, and generated PERK-cKO^Astrocyte^ mice [64]. They found that deletion of *Perk* in astrocytes increased morbidity and mortality after stroke. Moreover, since mounting evidence indicates that the PERK pathway is involved in cognitive functions (detailed below), they performed a battery of behavioral tests. However, they did not find any significant effect on learning, memory, or cognition in middle-aged and old PERK-cKO^Astrocyte^ mice [64]. These findings demonstrate that astrocytic PERK exerts a minimal influence on cognitive function, but plays a protective role in responding to neural injury. Notably, another study showed that global *Atf4* knockout mice had smaller infarcts and better neurologic outcomes after stroke [65]. This finding may support that the primary beneficial effects of PERK signaling in stroke could be attributed to an increase in p-eIF2α and a decrease in protein synthesis.

### 4.2. Experimental Evidence from Pharmacologic Tools 

ER stress has been implicated in various disease conditions, making it a promising therapeutic target. Consequently, many small molecule inhibitors and activators specific to the UPR, including the PERK branch, have been identified [9,66], constituting invaluable tools for translational research in stroke and other diseases associated with ER stress. The relevant experimental brain ischemia studies are listed in Table 1.

#### 4.2.1. PERK Inhibitors

Compounds GSK2606414 and GSK2656157 have been identified for their ability to inhibit PERK autophosphorylation, thereby preventing PERK activation [75,76]. These two compounds feature an induline core structure and interact with the PERK cytosolic kinase domain. However, both compounds can also inhibit RIPK1 and KIT, indicating their off-target effects. For example, these two compounds were found to completely inhibit TNF-mediated RIPK1-dependent cell death without affecting PERK activity in cells. Moreover, GSK2606414 can inhibit KIT tyrosine kinase activity and enhance KIT endocytosis and its lysosomal degradation [77,78]. Later, highly selective PERK inhibitors AMG52 and AMG44 were developed. But, compared to GSK2606414, they exhibit less potency in inhibiting the PERK pathway in cellular assays [79]. Among these inhibitors, GSK2606414, which has been proved to cross the BBB, has been assessed in stroke [67,80]. In a recent study, rats were subjected to 90 min MCAO, and after 3 h, treated with GSK2606414 via oral gavage at three different doses (low, medium, and high), followed by daily treatment for 4 days. Notably, rats in both the medium- and high-dose treatment groups exhibited smaller infarct size and performed better in various functional outcome tests, including neurologic scoring, the wire hanging test, the Morris water maze, and the novel object recognition test, compared to the vehicle group [67]. In this study, however, critical experimental details, especially regarding the surgical conditions and animal inclusion/exclusion criteria, were lacking. Moreover, given the well-known off-target effects of GSK2606414, whether its observed beneficial effects in stroke are due to inhibition of the PERK pathway requires further clarification. 

#### 4.2.2. PERK Activators

Two small molecules, CCT020312 and MK-28, are reportedly PERK activators [81,82]. A cell culture study demonstrated that CCT020312 selectively activated the PERK pathway without affecting the ATF6 or IRE1 UPR branches and, moreover, it failed to increase p-eIF2α in MEF cells from PERK^−/−^ mice, supporting its specificity [81]. MK-28 has been shown to increase PERK phosphorylation in cells [82]. Interestingly, both CCT020312 and MK-28 protected ST*Hdh*^Q111/11^ cells (a striatal cell line expressing mutant huntingtin) against ER stress-induced cell death, but did not show such protective effects in PERK^−/−^ cells [82]. However, their mechanisms of action that lead to PERK activation are still unknown and thus, potential off-target effects remain a concern. Nonetheless, acute treatment with CCT020312 has been examined recently in a stroke study [69]. This study investigated the effects of CCT020312 administered via intracerebroventricular injection in a rat model of 90 min MCAO. The data indicated that the treatment had no significant effect on the levels of BAX, CHOP, ATF4, and p-eIF2α, nor did it modulate infarct volumes. It is important to note that in this report, essential treatment information (e.g., timing of intervention) was not provided, and the animal sample sizes were small [69].

#### 4.2.3. eIF2α Phosphatase Inhibitors

Phosphorylation of eIF2α is the key step in PERK signaling under ER stress. However, when ER stress is resolved, dephosphorylation of p-eIF2α is necessary to ensure normal protein synthesis. This process is primarily mediated by CReP and GADD34, both of which can recruit a protein phosphatase 1 (PP1) catalytic subunit to form a holophosphatase complex that specifically dephosphorylates p-eIF2α [83,84]. Thus, one strategy for enhancing PERK signaling is to inhibit the activity of CReP and/or GADD34.

The first reported selective inhibitor of p-eIF2α dephosphorylation is salubrinal, which was discovered through screening for compounds that protected cells from ER stress-induced apoptosis [28]. Since its discovery, salubrinal has been widely used to study the impact of inhibiting p-eIF2α dephosphorylation under various conditions. Surprisingly, the mechanism and target of its action are still elusive [85]. Later, guanabenz and sephin-1 that can suppress translation recovery after ER stress, were proposed as selective GADD34 inhibitors [85,86,87]. However, their mechanisms of action are under debate [88,89]. Nevertheless, several studies have used salubrinal in brain ischemia/stroke research and found beneficial effects after acute treatment. One study demonstrated that intraperitoneal administration of salubrinal at 30 min before MCAO reduced infarct sizes at 24 h after stroke in rats (*n* = 5), although neurologic function was not evaluated [70]. Moreover, it has been shown that in a two-vessel occlusion rat model, treatment with salubrinal at 1 h and 24 h after a 15 min ischemia significantly reduced areas of selective neuronal loss at day 7 [72,73]. Notably, acute treatment with salubrinal can reverse worse stroke outcomes observed in PERK-cKO^Neuron^ mice [21]. Together, these data support that enhancing the PERK/eIF2α pathway during the acute phase is protective in brain ischemia. 

#### 4.2.4. eIF2B Activators

As discussed above, p-eIF2α-mediated suppression of protein synthesis results from its strong binding with eIF2B, which reduces TC formation for protein translation. One important small molecule targeting eIF2B is ISRIB, which can cross the BBB. Evidence indicates that ISRIB acts allosterically to stabilize an active conformation of eIF2B that is resistant to p-eIF2α, thus inhibiting the downstream effects of p-eIF2α and reversing translational attenuation [90]. Recently, compounds 2BAct and PRXS571 were synthesized as novel selective eIF2B activators with the ability to cross the BBB [91,92]. Moreover, 2BAct exhibits suitability for oral administration with diet, allowing for its simple long-term application [92]. Notably, it has been shown that ISRIB, 2BAct, and PRXS571 alleviate ER stress-induced translational inhibition without affecting ATF4 expression [91]. A recent rat study used a multiple treatment regimen to evaluate ISRIB in stroke, with the first dosing at 3 h post 90-minitue MCAO, followed by three additional doses with a 12 h interval between each dose. The authors found that ISRIB treatment improved stroke outcome, reflected in many parameters, including infarct volumes, muscle strength, motor function, and memory function [74]. However, it is noted that limited surgical information was provided in this report. Interestingly, ISRIB has shown beneficial effects in improving cognitive function, as discussed below.

## 5. The PERK Pathway in Memory, Aging, and Neurodegenerative Diseases

Compared to limited research in stroke, a large body of literature reports on the role of PERK in cognitive function and neurodegenerative diseases. Findings from these studies may have important implications for stroke research and, thus, are briefly discussed here.

It has long been established that protein synthesis is an essential step for memory consolidation [93]. Thus, it is not a surprise that the PERK/p-eIF2α pathway has been shown to play an important role in regulating the memory process. One study demonstrated that local inhibition of PERK activity by infusing a PERK inhibitor (GSK2606414) into the hippocampal CA1 region enhanced hippocampal-dependent memory [94]. Interestingly, the authors also found that PERK mRNA levels in the hippocampus were increased in 13-month vs. 4-month-old mice, and PERK silencing in the hippocampus reversed age-associated memory decline. Another study that used eIF2α^+/S51A^ mice, which express eIF2α(S51A) that cannot be phosphorylated due to a mutation of the eIF2α phosphorylation site, demonstrated that these mice exhibited enhancement in various cognitive tests, including spatial learning and memory, fear conditioning, and long-term taste memory [95]. Based on the same strategy, genetic ablation of p-eIF2α in excitatory or inhibitory neurons increased global translation and enhanced synaptic plasticity and long-term memory [96]. In line with the data from genetic manipulations, ISRIB-treated mice performed significantly better in the Morris water maze and contextual fear conditioning tests, compared to vehicle-treated mice [97]. Thus, inhibition of the PERK/eIF2α pathway appears to improve cognitive function in young and aged mice.

Samples from post-mortem patients and mouse models consistently reveal elevated levels of PERK, p-PERK, and p-eIF2α in various neurodegenerative diseases, including Alzheimer’s disease (AD), Parkinson’s disease, and Huntington’s disease. Thus, it is believed that the PERK/eIF2α pathway contributes to the development and progression of these diseases [11,12]. In this respect, many experimental AD studies on the PERK/eIF2α pathway have been performed [98]. In line with clinical observations, levels of p-eIF2α and phosphorylated PERK are increased in the brains of 5XFAD mice, an AD mouse model. When 5XFAD mice were crossed with heterozygous PERK knockout mice to reduce PERK protein levels, the PERK haploinsufficiency prevented an increase in β-site APP-cleaving enzyme 1 (BACE1), the rate-limiting enzyme for Aβ production. Consequently, Aβ peptides and plaque burden were reduced, and memory deficits were rescued in 5XFAD mice [99]. Similar results were obtained when PERK was genetically deleted in selected neurons in the AAP-PS1 AD mouse model [100]. Consistently, it has been shown that daily ISRIB treatment can restore synaptic plasticity and improve long-term memory in mouse AD models [101,102].

## 6. Concluding Remarks

After ischemic stroke, a cascade of pathologic processes is activated, including oxidative stress, ER stress, and mitochondrial dysfunction. Many of these processes can be regulated by the PERK pathway. Critically, experimental stroke studies have shown that either pharmacologic activation (e.g., salubrinal) or inhibition (e.g., GSK2606414 and ISRIB) of the PERK pathway can improve stroke outcomes. The underlying reasons for this discrepancy remain unknown. However, strong evidence obtained from genetic modulation of the PERK/p-eIF2α pathway supports the notion that activation of this pathway improves acute outcome after ischemic stroke [21,64]. Notably, no study has yet attempted to specifically dissect the role of the PERK pathway in stroke recovery during the chronic phase. In this respect, an intriguing perspective is the therapeutic potential of inhibiting the PERK/p-eIF2α pathway to enhance protein synthesis and promote brain remodeling, thus improving long-term functional recovery after stroke. For example, cognitive and memory impairment is common among stroke survivors, and greatly affects their quality of life [103,104]. Moreover, Suenaga et al. have shown that aged mice exhibited significantly worse long-term cognitive and memory deficits after stroke compared to young mice [105]. Given the beneficial effects of ISRIB in aged mice and neurodegenerative disease models, ISRIB treatment during the recovery phase holds promise for improving functional outcomes after a stroke. It is important to note, however, that complete or long-term inhibition of PERK by pharmacologic approaches could be toxic. It is known that activation of the PERK/p-eIF2α pathway is essential for the pancreas to cope with constant ER stress caused by a high load of secretory protein synthesis [106]. Thus, using an optimized dosage of PERK/p-eIF2α pathway inhibitors for chronic treatment is crucial. Indeed, in one study, long-term daily systemic administration of low-dose ISRIB showed therapeutic effects in AD mice without inducing toxic side effects [101]. Taken together, the PERK/p-eIF2α pathway emerges as a promising therapeutic target for strokes, by reducing brain damage during the acute phase and improving neurologic recovery during the chronic phase.

## Figures and Tables

**Table 1:** Brain ischemia studies with pharmacologic interventions targeting the PERK pathway.

Compound	Animal	Ischemia Model	Dose	Intervention Regimen	Route	Outcome	Reference
*PERK inhibitors*							
GSK2606414	Rats	tMCAO	50, 100, or 150 mg/kg	Starting at 3 h post-MCAO, followed by 4 doses at a 24 h interval	Oral	Improved	[67]
	Mice	tMCAO	4 μL (20 μM)	24 h before tMCAO	ICV	No effect	[68]
*PERK activator*							
CCT020312	SD rats	tMCAO	Unspecified	Unspecified	ICV	No effect	[69]
*eIF2α phosphatase inhibitor*							
Salubrinal	Mice	tMCAO	1 mg/kg	30 min after reperfusion	IP	Improved	[21]
	SD rats	tMCAO	1 mg/kg	30 min before tMCAO	IP	Improved	[70]
	SD rats	GCI	1 mg/kg	1 h and 25 h after GCI	IP	Improved	[71]
	SD rats	GCI	1 mg/kg	1 h and 24 h after GCI	IP	Improved	[72]
	Rats	GCI	1 mg/kg	1 h and 24 h after GCI	IP	Improved	[73]
*eIF2B activator*							
ISRIB	Wistar rats	tMCAO	2.5 mg/kg	3 h post MCAO followed by 3 doses at a 12 h interval	IP	Improved	[74]

SD: Sprague–Dawley; GCI: global cerebral ischemia; h: hour; min: minute; IP: intraperitoneal injection; ICV: intracerebroventricular injection.

## Data Availability

Data sharing is not applicable.

## References

[1] Martin S.S., Aday A.W., Almarzooq Z.I., Anderson C.A.M., Arora P., Avery C.L., Baker-Smith C.M., Barone Gibbs B., Beaton A.Z., Boehme A.K. (2024). 2024 Heart Disease and Stroke Statistics: A Report of US and Global Data from the American Heart Association. Circulation.

[2] Hetz C., Zhang K., Kaufman R.J. (2020). Mechanisms, regulation and functions of the unfolded protein response. Nat. Rev. Mol. Cell Biol..

[3] Wang Z., Yang W. (2019). Impaired capacity to restore proteostasis in the aged brain after ischemia: Implications for translational brain ischemia research. Neurochem. Int..

[4] Yang W., Paschen W. (2016). Unfolded protein response in brain ischemia: A timely update. J. Cereb. Blood Flow. Metab..

[5] Li X., Yang W. (2021). An update on the unfolded protein response in brain ischemia: Experimental evidence and therapeutic opportunities. Neurochem. Int..

[6] Wang L., Liu Y., Zhang X., Ye Y., Xiong X., Zhang S., Gu L., Jian Z., Wang H. (2022). Endoplasmic Reticulum Stress and the Unfolded Protein Response in Cerebral Ischemia/Reperfusion Injury. Front. Cell. Neurosci..

[7] Iurlaro R., Munoz-Pinedo C. (2016). Cell death induced by endoplasmic reticulum stress. FEBS J..

[8] Costa-Mattioli M., Walter P. (2020). The integrated stress response: From mechanism to disease. Science.

[9] Marciniak S.J., Chambers J.E., Ron D. (2022). Pharmacological targeting of endoplasmic reticulum stress in disease. Nat. Rev. Drug Discov..

[10] Lin J.H., Li H., Yasumura D., Cohen H.R., Zhang C., Panning B., Shokat K.M., Lavail M.M., Walter P. (2007). IRE1 signaling affects cell fate during the unfolded protein response. Science.

[11] Almeida L.M., Pinho B.R., Duchen M.R., Oliveira J.M.A. (2022). The PERKs of mitochondria protection during stress: Insights for PERK modulation in neurodegenerative and metabolic diseases. Biol. Rev. Camb. Philos. Soc..

[12] Talukdar G., Orr H.T., Lei Z. (2023). The PERK pathway: Beneficial or detrimental for neurodegenerative diseases and tumor growth and cancer. Hum. Mol. Genet..

[13] Wang Z.V., Deng Y., Gao N., Pedrozo Z., Li D.L., Morales C.R., Criollo A., Luo X., Tan W., Jiang N. (2014). Spliced X-box binding protein 1 couples the unfolded protein response to hexosamine biosynthetic pathway. Cell.

[14] Jiang M., Yu S., Yu Z., Sheng H.X., Li Y., Liu S., Warner D.S., Paschen W., Yang W. (2017). XBP1 (X-Box-Binding Protein-1)-Dependent O-GlcNAcylation Is Neuroprotective in Ischemic Stroke in Young Mice and Its Impairment in Aged Mice Is Rescued by Thiamet-G. Stroke.

[15] Wang Z., Li X., Spasojevic I., Lu L., Shen Y., Qu X., Hoffmann U., Warner D.S., Paschen W., Sheng H. (2021). Increasing O-GlcNAcylation is neuroprotective in young and aged brains after ischemic stroke. Exp. Neurol..

[16] Fahie K.M.M., Papanicolaou K.N., Zachara N.E. (2022). Integration of O-GlcNAc into Stress Response Pathways. Cells.

[17] Yoshikawa A., Kamide T., Hashida K., Ta H.M., Inahata Y., Takarada-Iemata M., Hattori T., Mori K., Takahashi R., Matsuyama T. (2015). Deletion of Atf6alpha impairs astroglial activation and enhances neuronal death following brain ischemia in mice. J. Neurochem..

[18] Yu Z., Sheng H., Liu S., Zhao S., Glembotski C.C., Warner D.S., Paschen W., Yang W. (2017). Activation of the ATF6 branch of the unfolded protein response in neurons improves stroke outcome. J. Cereb. Blood Flow. Metab..

[19] Li X., Li R., Lu L., Dhar A., Sheng H., Yang W. (2022). Beneficial effects of neuronal ATF6 activation in permanent ischemic stroke. Front. Cell. Neurosci..

[20] Wang Z., Li Q., Kolls B.J., Mace B., Yu S., Li X., Liu W., Chaparro E., Shen Y., Dang L. (2023). Sustained overexpression of spliced X-box-binding protein-1 in neurons leads to spontaneous seizures and sudden death in mice. Commun. Biol..

[21] Wang Y.C., Li X., Shen Y., Lyu J., Sheng H., Paschen W., Yang W. (2020). PERK (Protein Kinase RNA-Like ER Kinase) Branch of the Unfolded Protein Response Confers Neuroprotection in Ischemic Stroke by Suppressing Protein Synthesis. Stroke.

[22] Shen Y., Yan B., Zhao Q., Wang Z., Wu J., Ren J., Wang W., Yu S., Sheng H., Crowley S.D. (2018). Aging is associated with impaired activation of protein homeostasis-related pathways after cardiac arrest in mice. J. Am. Heart Assoc..

[23] Liu S., Sheng H., Yu Z., Paschen W., Yang W. (2016). O-linked beta-N-acetylglucosamine modification of proteins is activated in post-ischemic brains of young but not aged mice: Implications for impaired functional recovery from ischemic stress. J. Cereb. Blood Flow. Metab..

[24] Owen C.R., Kumar R., Zhang P., McGrath B.C., Cavener D.R., Krause G.S. (2005). PERK is responsible for the increased phosphorylation of eIF2alpha and the severe inhibition of protein synthesis after transient global brain ischemia. J. Neurochem..

[25] Hinnebusch A.G., Lorsch J.R. (2012). The mechanism of eukaryotic translation initiation: New insights and challenges. Cold Spring Harb. Perspect. Biol..

[26] Ron D., Walter P. (2007). Signal integration in the endoplasmic reticulum unfolded protein response. Nat. Rev. Mol. Cell Biol..

[27] Harding H.P., Zhang Y., Bertolotti A., Zeng H., Ron D. (2000). Perk is essential for translational regulation and cell survival during the unfolded protein response. Mol. Cell.

[28] Boyce M., Bryant K.F., Jousse C., Long K., Harding H.P., Scheuner D., Kaufman R.J., Ma D., Coen D.M., Ron D. (2005). A selective inhibitor of eIF2alpha dephosphorylation protects cells from ER stress. Science.

[29] Raben N., Puertollano R. (2016). TFEB and TFE3: Linking Lysosomes to Cellular Adaptation to Stress. Annu. Rev. Cell Dev. Biol..

[30] Martina J.A., Diab H.I., Brady O.A., Puertollano R. (2016). TFEB and TFE3 are novel components of the integrated stress response. EMBO J..

[31] Kim H.J., Joe Y., Rah S.Y., Kim S.K., Park S.U., Park J., Kim J., Ryu J., Cho G.J., Surh Y.J. (2018). Carbon monoxide induced TFEB nuclear translocation enhances mitophagy/mitochondrial biogenesis in hepatocytes and ameliorates inflammatory liver injury. Cell Death Dis..

[32] Creamer T.P. (2020). Calcineurin. Cell Commun. Signal..

[33] Wang X., Fang Y., Huang Q., Xu P., Lenahan C., Lu J., Zheng J., Dong X., Shao A., Zhang J. (2021). An updated review of autophagy in ischemic stroke: From mechanisms to therapies. Exp. Neurol..

[34] Ong G., Logue S.E. (2023). Unfolding the Interactions between Endoplasmic Reticulum Stress and Oxidative Stress. Antioxidants.

[35] Wang L., Zhang X., Xiong X., Zhu H., Chen R., Zhang S., Chen G., Jian Z. (2022). Nrf2 Regulates Oxidative Stress and Its Role in Cerebral Ischemic Stroke. Antioxidants.

[36] Cullinan S.B., Gordan J.D., Jin J., Harper J.W., Diehl J.A. (2004). The Keap1-BTB protein is an adaptor that bridges Nrf2 to a Cul3-based E3 ligase: Oxidative stress sensing by a Cul3-Keap1 ligase. Mol. Cell. Biol..

[37] He C.H., Gong P., Hu B., Stewart D., Choi M.E., Choi A.M., Alam J. (2001). Identification of activating transcription factor 4 (ATF4) as an Nrf2-interacting protein. Implication for heme oxygenase-1 gene regulation. J. Biol. Chem..

[38] Shah Z.A., Li R.C., Thimmulappa R.K., Kensler T.W., Yamamoto M., Biswal S., Dore S. (2007). Role of reactive oxygen species in modulation of Nrf2 following ischemic reperfusion injury. Neuroscience.

[39] Panahian N., Yoshiura M., Maines M.D. (1999). Overexpression of heme oxygenase-1 is neuroprotective in a model of permanent middle cerebral artery occlusion in transgenic mice. J. Neurochem..

[40] Jiang R.Q., Li Q.Q., Sheng R. (2023). Mitochondria associated ER membranes and cerebral ischemia: Molecular mechanisms and therapeutic strategies. Pharmacol. Res..

[41] Prinz W.A., Toulmay A., Balla T. (2020). The functional universe of membrane contact sites. Nat. Rev. Mol. Cell Biol..

[42] Verfaillie T., Rubio N., Garg A.D., Bultynck G., Rizzuto R., Decuypere J.P., Piette J., Linehan C., Gupta S., Samali A. (2012). PERK is required at the ER-mitochondrial contact sites to convey apoptosis after ROS-based ER stress. Cell Death Differ..

[43] Fiorese C.J., Schulz A.M., Lin Y.F., Rosin N., Pellegrino M.W., Haynes C.M. (2016). The Transcription Factor ATF5 Mediates a Mammalian Mitochondrial UPR. Curr. Biol..

[44] Zhou D., Palam L.R., Jiang L., Narasimhan J., Staschke K.A., Wek R.C. (2008). Phosphorylation of eIF2 directs ATF5 translational control in response to diverse stress conditions. J. Biol. Chem..

[45] Gomes L.C., Di Benedetto G., Scorrano L. (2011). During autophagy mitochondria elongate, are spared from degradation and sustain cell viability. Nat. Cell Biol..

[46] Balsa E., Soustek M.S., Thomas A., Cogliati S., Garcia-Poyatos C., Martin-Garcia E., Jedrychowski M., Gygi S.P., Enriquez J.A., Puigserver P. (2019). ER and Nutrient Stress Promote Assembly of Respiratory Chain Supercomplexes through the PERK-eIF2alpha Axis. Mol. Cell.

[47] Lebeau J., Saunders J.M., Moraes V.W.R., Madhavan A., Madrazo N., Anthony M.C., Wiseman R.L. (2018). The PERK Arm of the Unfolded Protein Response Regulates Mitochondrial Morphology during Acute Endoplasmic Reticulum Stress. Cell Rep..

[48] Scorrano L., Ashiya M., Buttle K., Weiler S., Oakes S.A., Mannella C.A., Korsmeyer S.J. (2002). A distinct pathway remodels mitochondrial cristae and mobilizes cytochrome c during apoptosis. Dev. Cell.

[49] Latorre-Muro P., O’Malley K.E., Bennett C.F., Perry E.A., Balsa E., Tavares C.D.J., Jedrychowski M., Gygi S.P., Puigserver P. (2021). A cold-stress-inducible PERK/OGT axis controls TOM70-assisted mitochondrial protein import and cristae formation. Cell Metab..

[50] Sassano M.L., van Vliet A.R., Vervoort E., Van Eygen S., Van den Haute C., Pavie B., Roels J., Swinnen J.V., Spinazzi M., Moens L. (2023). PERK recruits E-Syt1 at ER-mitochondria contacts for mitochondrial lipid transport and respiration. J. Cell Biol..

[51] McCullough K.D., Martindale J.L., Klotz L.O., Aw T.Y., Holbrook N.J. (2001). Gadd153 sensitizes cells to endoplasmic reticulum stress by down-regulating Bcl2 and perturbing the cellular redox state. Mol. Cell. Biol..

[52] Puthalakath H., O’Reilly L.A., Gunn P., Lee L., Kelly P.N., Huntington N.D., Hughes P.D., Michalak E.M., McKimm-Breschkin J., Motoyama N. (2007). ER stress triggers apoptosis by activating BH3-only protein Bim. Cell.

[53] Marciniak S.J., Yun C.Y., Oyadomari S., Novoa I., Zhang Y., Jungreis R., Nagata K., Harding H.P., Ron D. (2004). CHOP induces death by promoting protein synthesis and oxidation in the stressed endoplasmic reticulum. Genes Dev..

[54] Novoa I., Zhang Y., Zeng H., Jungreis R., Harding H.P., Ron D. (2003). Stress-induced gene expression requires programmed recovery from translational repression. EMBO J..

[55] Brush M.H., Weiser D.C., Shenolikar S. (2003). Growth arrest and DNA damage-inducible protein GADD34 targets protein phosphatase 1 alpha to the endoplasmic reticulum and promotes dephosphorylation of the alpha subunit of eukaryotic translation initiation factor 2. Mol. Cell. Biol..

[56] Hetz C. (2012). The unfolded protein response: Controlling cell fate decisions under ER stress and beyond. Nat. Rev. Mol. Cell Biol..

[57] Tajiri S., Oyadomari S., Yano S., Morioka M., Gotoh T., Hamada J.I., Ushio Y., Mori M. (2004). Ischemia-induced neuronal cell death is mediated by the endoplasmic reticulum stress pathway involving CHOP. Cell Death Differ..

[58] Han J., Back S.H., Hur J., Lin Y.H., Gildersleeve R., Shan J., Yuan C.L., Krokowski D., Wang S., Hatzoglou M. (2013). ER-stress-induced transcriptional regulation increases protein synthesis leading to cell death. Nat. Cell Biol..

[59] B’Chir W., Maurin A.C., Carraro V., Averous J., Jousse C., Muranishi Y., Parry L., Stepien G., Fafournoux P., Bruhat A. (2013). The eIF2alpha/ATF4 pathway is essential for stress-induced autophagy gene expression. Nucleic Acids Res..

[60] Liu K., Zhao C., Adajar R.C., DeZwaan-McCabe D., Thomas Rutkowski D. (2024). A beneficial adaptive role for CHOP in driving cell fate selection during ER stress. EMBO Rep..

[61] Li H.Q., Xia S.N., Xu S.Y., Liu P.Y., Gu Y., Bao X.Y., Xu Y., Cao X. (2021). γ-Glutamylcysteine Alleviates Ischemic Stroke-Induced Neuronal Apoptosis by Inhibiting ROS-Mediated Endoplasmic Reticulum Stress. Oxidative Med. Cell. Longev..

[62] Wu Y., Fan X., Chen S., Deng L., Jiang L., Yang S., Dong Z. (2022). Geraniol-Mediated Suppression of Endoplasmic Reticulum Stress Protects against Cerebral Ischemia-Reperfusion Injury via the PERK-ATF4-CHOP Pathway. Int. J. Mol. Sci..

[63] Nakka V.P., Gogada R., Simhadri P.K., Qadeer M.A., Phanithi P.B. (2022). Post-treatment with apocynin at a lower dose regulates the UPR branch of eIF2α and XBP-1 pathways after stroke. Brain Res. Bull..

[64] Lahiri A., Walton J.C., Zhang N., Billington N., DeVries A.C., Meares G.P. (2023). Astrocytic deletion of protein kinase R-like ER kinase (PERK) does not affect learning and memory in aged mice but worsens outcome from experimental stroke. J. Neurosci. Res..

[65] Lange P.S., Chavez J.C., Pinto J.T., Coppola G., Sun C.W., Townes T.M., Geschwind D.H., Ratan R.R. (2008). ATF4 is an oxidative stress-inducible, prodeath transcription factor in neurons in vitro and in vivo. J. Exp. Med..

[66] Hetz C., Axten J.M., Patterson J.B. (2019). Pharmacological targeting of the unfolded protein response for disease intervention. Nat. Chem. Biol..

[67] Dhir N., Jain A., Sharma A.R., Prakash A., Radotra B.D., Medhi B. (2023). PERK inhibitor, GSK2606414, ameliorates neuropathological damage, memory and motor functional impairments in cerebral ischemia via PERK/p-eIF2α/ATF4/CHOP signaling. Metab. Brain Dis..

[68] Li Y., Zhang Y., Fu H., Huang H., Lu Q., Qin H., Wu Y., Huang H., Mao G., Wei Z. (2020). Hes1 Knockdown Exacerbates Ischemic Stroke Following tMCAO by Increasing ER Stress-Dependent Apoptosis via the PERK/eIF2α/ATF4/CHOP Signaling Pathway. Neurosci. Bull..

[69] Fei H., Xiang P., Luo W., Tan X., Gu C., Liu M., Chen M., Wang Q., Yang J. (2021). CTRP1 Attenuates Cerebral Ischemia/Reperfusion Injury via the PERK Signaling Pathway. Front. Cell Dev. Biol..

[70] Nakka V.P., Gusain A., Raghubir R. (2010). Endoplasmic reticulum stress plays critical role in brain damage after cerebral ischemia/reperfusion in rats. Neurotox. Res..

[71] Anuncibay-Soto B., Perez-Rodriguez D., Santos-Galdiano M., Font E., Regueiro-Purrinos M., Fernandez-Lopez A. (2016). Post-ischemic salubrinal treatment results in a neuroprotective role in global cerebral ischemia. J. Neurochem..

[72] Anuncibay-Soto B., Perez-Rodriguez D., Santos-Galdiano M., Font-Belmonte E., Ugidos I.F., Gonzalez-Rodriguez P., Regueiro-Purrinos M., Fernandez-Lopez A. (2018). Salubrinal and robenacoxib treatment after global cerebral ischemia. Exploring the interactions between ER stress and inflammation. Biochem. Pharmacol..

[73] Font-Belmonte E., Ugidos I.F., Santos-Galdiano M., Gonzalez-Rodriguez P., Anuncibay-Soto B., Perez-Rodriguez D., Gonzalo-Orden J.M., Fernandez-Lopez A. (2019). Post-ischemic salubrinal administration reduces necroptosis in a rat model of global cerebral ischemia. J. Neurochem..

[74] Dhir N., Jain A., Sharma A.R., Sharma S., Mahendru D., Patial A., Malik D., Prakash A., Attri S.V., Bhattacharyya S. (2023). Rat BM-MSCs secretome alone and in combination with stiripentol and ISRIB, ameliorated microglial activation and apoptosis in experimental stroke. Behav. Brain Res..

[75] Axten J.M., Romeril S.P., Shu A., Ralph J., Medina J.R., Feng Y., Li W.H., Grant S.W., Heerding D.A., Minthorn E. (2013). Discovery of GSK2656157: An Optimized PERK Inhibitor Selected for Preclinical Development. ACS Med. Chem. Lett..

[76] Axten J.M., Medina J.R., Feng Y., Shu A., Romeril S.P., Grant S.W., Li W.H., Heerding D.A., Minthorn E., Mencken T. (2012). Discovery of 7-methyl-5-(1-{[3-(trifluoromethyl)phenyl]acetyl}-2,3-dihydro-1H-indol-5-yl)-7H-pyrrolo[2,3-d]pyrimidin-4-amine (GSK2606414), a potent and selective first-in-class inhibitor of protein kinase R (PKR)-like endoplasmic reticulum kinase (PERK). J. Med. Chem..

[77] Rojas-Rivera D., Delvaeye T., Roelandt R., Nerinckx W., Augustyns K., Vandenabeele P., Bertrand M.J.M. (2017). When PERK inhibitors turn out to be new potent RIPK1 inhibitors: Critical issues on the specificity and use of GSK2606414 and GSK2656157. Cell Death Differ..

[78] Mahameed M., Wilhelm T., Darawshi O., Obiedat A., Tommy W.S., Chintha C., Schubert T., Samali A., Chevet E., Eriksson L.A. (2019). The unfolded protein response modulators GSK2606414 and KIRA6 are potent KIT inhibitors. Cell Death Dis..

[79] Smith A.L., Andrews K.L., Beckmann H., Bellon S.F., Beltran P.J., Booker S., Chen H., Chung Y.A., D’Angelo N.D., Dao J. (2015). Discovery of 1H-pyrazol-3(2H)-ones as potent and selective inhibitors of protein kinase R-like endoplasmic reticulum kinase (PERK). J. Med. Chem..

[80] Moreno J.A., Halliday M., Molloy C., Radford H., Verity N., Axten J.M., Ortori C.A., Willis A.E., Fischer P.M., Barrett D.A. (2013). Oral treatment targeting the unfolded protein response prevents neurodegeneration and clinical disease in prion-infected mice. Sci. Transl. Med..

[81] Stockwell S.R., Platt G., Barrie S.E., Zoumpoulidou G., Te Poele R.H., Aherne G.W., Wilson S.C., Sheldrake P., McDonald E., Venet M. (2012). Mechanism-based screen for G1/S checkpoint activators identifies a selective activator of EIF2AK3/PERK signalling. PLoS ONE.

[82] Ganz J., Shacham T., Kramer M., Shenkman M., Eiger H., Weinberg N., Iancovici O., Roy S., Simhaev L., Da’adoosh B. (2020). A novel specific PERK activator reduces toxicity and extends survival in Huntington’s disease models. Sci. Rep..

[83] Novoa I., Zeng H., Harding H.P., Ron D. (2001). Feedback inhibition of the unfolded protein response by GADD34-mediated dephosphorylation of eIF2alpha. J. Cell Biol..

[84] Jousse C., Oyadomari S., Novoa I., Lu P., Zhang Y., Harding H.P., Ron D. (2003). Inhibition of a constitutive translation initiation factor 2alpha phosphatase, CReP, promotes survival of stressed cells. J. Cell Biol..

[85] Carrara M., Sigurdardottir A., Bertolotti A. (2017). Decoding the selectivity of eIF2alpha holophosphatases and PPP1R15A inhibitors. Nat. Struct. Mol. Biol..

[86] Das I., Krzyzosiak A., Schneider K., Wrabetz L., D’Antonio M., Barry N., Sigurdardottir A., Bertolotti A. (2015). Preventing proteostasis diseases by selective inhibition of a phosphatase regulatory subunit. Science.

[87] Tsaytler P., Harding H.P., Ron D., Bertolotti A. (2011). Selective inhibition of a regulatory subunit of protein phosphatase 1 restores proteostasis. Science.

[88] Crespillo-Casado A., Chambers J.E., Fischer P.M., Marciniak S.J., Ron D. (2017). PPP1R15A-mediated dephosphorylation of eIF2α is unaffected by Sephin1 or Guanabenz. eLife.

[89] Crespillo-Casado A., Claes Z., Choy M.S., Peti W., Bollen M., Ron D. (2018). A Sephin1-insensitive tripartite holophosphatase dephosphorylates translation initiation factor 2α. J. Biol. Chem..

[90] Zyryanova A.F., Kashiwagi K., Rato C., Harding H.P., Crespillo-Casado A., Perera L.A., Sakamoto A., Nishimoto M., Yonemochi M., Shirouzu M. (2021). ISRIB Blunts the Integrated Stress Response by Allosterically Antagonising the Inhibitory Effect of Phosphorylated eIF2 on eIF2B. Mol. Cell.

[91] Marlin E., Valencia M., Peregrín N., Ferrero R., Nicolás M.J., Vinueza-Gavilanes R., Pineda-Lucena A., Artieda J., Arrasate M., Aragón T. (2023). Pharmacological inhibition of the integrated stress response accelerates disease progression in an amyotrophic lateral sclerosis mouse model. Br. J. Pharmacol..

[92] Wong Y.L., LeBon L., Basso A.M., Kohlhaas K.L., Nikkel A.L., Robb H.M., Donnelly-Roberts D.L., Prakash J., Swensen A.M., Rubinstein N.D. (2019). eIF2B activator prevents neurological defects caused by a chronic integrated stress response. eLife.

[93] Shrestha P., Klann E. (2022). Spatiotemporally resolved protein synthesis as a molecular framework for memory consolidation. Trends Neurosci..

[94] Sharma V., Ounallah-Saad H., Chakraborty D., Hleihil M., Sood R., Barrera I., Edry E., Kolatt Chandran S., Ben Tabou de Leon S., Kaphzan H. (2018). Local Inhibition of PERK Enhances Memory and Reverses Age-Related Deterioration of Cognitive and Neuronal Properties. J. Neurosci..

[95] Costa-Mattioli M., Gobert D., Stern E., Gamache K., Colina R., Cuello C., Sossin W., Kaufman R., Pelletier J., Rosenblum K. (2007). eIF2α phosphorylation bidirectionally regulates the switch from short- to long-term synaptic plasticity and memory. Cell.

[96] Sharma V., Sood R., Khlaifia A., Eslamizade M.J., Hung T.Y., Lou D., Asgarihafshejani A., Lalzar M., Kiniry S.J., Stokes M.P. (2020). eIF2α controls memory consolidation via excitatory and somatostatin neurons. Nature.

[97] Sidrauski C., Acosta-Alvear D., Khoutorsky A., Vedantham P., Hearn B.R., Li H., Gamache K., Gallagher C.M., Ang K.K., Wilson C. (2013). Pharmacological brake-release of mRNA translation enhances cognitive memory. eLife.

[98] Shacham T., Patel C., Lederkremer G.Z. (2021). PERK Pathway and Neurodegenerative Disease: To Inhibit or to Activate?. Biomolecules.

[99] Devi L., Ohno M. (2014). PERK mediates eIF2alpha phosphorylation responsible for BACE1 elevation, CREB dysfunction and neurodegeneration in a mouse model of Alzheimer’s disease. Neurobiol. Aging.

[100] Ma T., Trinh M.A., Wexler A.J., Bourbon C., Gatti E., Pierre P., Cavener D.R., Klann E. (2013). Suppression of eIF2alpha kinases alleviates Alzheimer’s disease-related plasticity and memory deficits. Nat. Neurosci..

[101] Oliveira M.M., Lourenco M.V., Longo F., Kasica N.P., Yang W., Ureta G., Ferreira D.D.P., Mendonca P.H.J., Bernales S., Ma T. (2021). Correction of eIF2-dependent defects in brain protein synthesis, synaptic plasticity, and memory in mouse models of Alzheimer’s disease. Sci. Signal..

[102] Hu Z., Yu P., Zhang Y., Yang Y., Zhu M., Qin S., Xu J.T., Duan D., Wu Y., Wang D. (2022). Inhibition of the ISR abrogates mGluR5-dependent long-term depression and spatial memory deficits in a rat model of Alzheimer’s disease. Transl. Psychiatry.

[103] Pollock A., St George B., Fenton M., Firkins L. (2012). Top ten research priorities relating to life after stroke. Lancet Neurol..

[104] Leys D., Henon H., Mackowiak-Cordoliani M.-A., Pasquier F. (2005). Poststroke dementia. Lancet Neurol..

[105] Suenaga J., Hu X., Pu H., Shi Y., Hassan S.H., Xu M., Leak R.K., Stetler R.A., Gao Y., Chen J. (2015). White matter injury and microglia/macrophage polarization are strongly linked with age-related long-term deficits in neurological function after stroke. Exp. Neurol..

[106] Harding H.P., Zeng H.Q., Zhang Y.H., Jungries R., Chung P., Plesken H., Sabatini D.D., Ron D. (2001). Diabetes mellitus and exocrine pancreatic dysfunction in Perk^−/−^ mice reveals a role for translational control in secretory cell survival. Mol. Cell.

